# Re-Evaluating Rapid Tranquillisation Practices in Elderly Patients (over 65 Years of Age) at a General Hospital: A Follow-Up Audit

**DOI:** 10.1192/bjo.2024.634

**Published:** 2024-08-01

**Authors:** Hardeep Singh, Harrison Wrench, Josie Jenkinson

**Affiliations:** Surrey and Borders NHS Foundation Trust, Surrey, United Kingdom

## Abstract

**Aims:**

This re-audit of rapid tranquillisation (RT) practices in patients over the age of 65 at a district general hospital took place as part of a wider quality improvement project to assess whether practices had improved following previous audits.

**Methods:**

Data was accessed using the hospital's electronic patient record system. Drug charts for patients over 65 admitted to six wards (total n = 172) were reviewed. The wards comprised three geriatric wards, two medical wards, and one surgical ward. Drug charts were reviewed using the audit tool developed in previous audits, which has been designed to collect relevant data according to the recognised standard (in this case the local mental health trust's RT guidance). Data was collected on RT type, RT frequency of RT, RT route, indication documentation, post-RT monitoring, nature of prescription (PRN, stat, or regular), underlying diagnosis of delirium or dementia, and involvement of specialist teams.

**Results:**

- Of the 172 audited patients, 9 (5.2%) received RT, compared with 13 out of 297 (4.3%) in the previous 2022 audit.
- PRN remained the most common prescription pattern, with two designated as stat and the remaining three mostly stat but occasionally incorporating PRN. Intramuscular administration continued to be the most common route in both cycles.
- In the current cycle, the maximum frequency was indicated in 55.5% of cases, whereas it was not indicated in the previous cycle.
- In the current cycle, indications were documented for 88.8% of prescriptions, a significant increase from 50% in the previous cycle. Furthermore, there was almost 100% compliance in nursing/medical documentation of RT administration in patient notes, which was lacking in the previous audit.
- Psych liaison or dementia team involvement was observed in around 33% of cases in the current cycle, whereas it was not evident in the previous cycle.
- Post-sedation monitoring in line with policy was not evident in either cycle.

**Conclusion:**

Overall, both audits highlighted consistent challenges in prescription practices and post-administration monitoring, albeit with variations in compliance levels and team involvement. Since the completion of this re-audit, a new RT policy has been approved which has much clearer guidance for the general hospital. This RT policy will be launched with a programme of teaching and training for the hospital. We aim to track progress by conducting a re-audit within 6–12 months.

